# *BnSIP1-1*, a Trihelix Family Gene, Mediates Abiotic Stress Tolerance and ABA Signaling in *Brassica napus*

**DOI:** 10.3389/fpls.2017.00044

**Published:** 2017-01-26

**Authors:** Junling Luo, Shaohua Tang, Fengling Mei, Xiaojue Peng, Jun Li, Xiaofei Li, Xiaohong Yan, Xinhua Zeng, Fang Liu, Yuhua Wu, Gang Wu

**Affiliations:** ^1^Oil Crops Research Institute of the Chinese Academy of Agricultural Sciences, Key Laboratory of Biology and Genetic Improvement of Oil Crops, Ministry of AgricultureWuhan, China; ^2^Key Laboratory of Molecular Biology and Gene Engineering of Jiangxi Province, College of Life Science, Nanchang UniversityNanchang, China

**Keywords:** *Brassica napus*, ABA, osmotic stress, salt, *BnSIP1-1*, trihelix gene family

## Abstract

The trihelix family genes have important functions in light-relevant and other developmental processes, but their roles in response to adverse environment are largely unclear. In this study, we identified a new gene, *BnSIP1-1*, which fell in the SIP1 (6b INTERACTING PROTEIN1) clade of the trihelix family with two trihelix DNA binding domains and a fourth amphipathic α-helix. BnSIP1-1 protein specifically targeted to the nucleus, and its expression can be induced by abscisic acid (ABA) and different stresses. Overexpression of *BnSIP1-1* improved seed germination under osmotic pressure, salt, and ABA treatments. Moreover, *BnSIP1-1* decreased the susceptibility of transgenic seedlings to osmotic pressure and ABA treatments, whereas there was no difference under salt stress between the transgenic and wild-type seedlings. ABA level in the transgenic seedlings leaves was higher than those in the control plants under normal condition. Under exogenous ABA treatment and mannitol stress, the accumulation of ABA in the transgenic plants was higher than that in the control plants; while under salt stress, the difference of ABA content before treatment was gradually smaller with the prolongation of salt treatment time, then after 24 h of treatment the ABA level was similar in transgenic and wild-type plants. The transcription levels of several general stress marker genes (*BnRD29A*, *BnERD15*, and *BnLEA1*) were higher in the transgenic plants than the wild-type plants, whereas salt-responsive genes (*BnSOS1*, *BnNHX1*, and *BnHKT*) were not significantly different or even reduced compared with the wild-type plants, which indicated that *BnSIP1-1* specifically exerted different regulatory mechanisms on the osmotic- and salt-response pathways in seedling period. Overall, these findings suggested that *BnSIP1-1* played roles in ABA synthesis and signaling, salt and osmotic stress response. To date, information about the involvement of the *Brassica napus* trihelix gene in abiotic response is scarce. Here, we firstly reported abiotic stress response and possible function mechanisms of a new trihelix gene in *B. napus*.

## Introduction

The trihelix transcription factor family is named after its conserved DNA binding domain which contains three tandem helices (helix-loop-helix-loop-helix). Phylogenetic analysis has identified several classes of trihelix transcription factors based on these features, including the GT-1, GT-2, SH4, GTγ and SIP1 subfamilies (Kaplan-Levy et al., 2012). The trihelix transcription factor family has 28 members in the *Arabidopsis* genome (Riechmann et al., 2000) and 22 members in the rice genome (Riano-Pachon et al., 2007). To date, there is no report about members of the trihelix family in rapeseeds (*Brassica napus L.*).

Early studies on the trihelix family focused only on their functions in the regulation of light-responsive genes (Gilmartin et al., 1992; Perisic and Lam, 1992; Kaplan-Levy et al., 2012). However, recent studies indicated that the trihelix family also played important roles in growth and developmental processes and in response to abiotic and biotic stresses (Li et al., 2008, 2015; Kaplan-Levy et al., 2012, 2014; Song et al., 2016; Wang et al., 2016; Zheng et al., 2016). Abiotic stresses affects plant growth and development and limits the productivity of crops (Boyer, 1982). To cope with various environmental stresses, plants have evolved various adjustment mechanisms to defend and adapt to stressful conditions, including the accumulation of osmoprotectants and the activation of stress-responsive pathways (Xiong et al., 2002). Several trihelix transcription factors have been shown to participate in the abiotic stress response (Kaplan-Levy et al., 2012; Qin et al., 2014). For example, Xie et al. (2009) showed that the constitutive expression of either *GmGT-2A* or *GmGT-2B* improved plant tolerance to salt, freezing and osmotic stress in transgenic *Arabidopsis* plants. Furthermore, other reports showed that the heterologous expression of *GT-3b* (from soybean) or *GT*γ*-1-3-like1* (from rice) improved osmotic stress tolerance in *Arabidopsis* (Park et al., 2004; Fang et al., 2010), while the heterologous expression of *PtaGTL1* (from *Poplar*) in *Arabidopsis* also enhanced the plants’ tolerance to drought (Weng et al., 2012). One member GT-4 was identified to improve salt tolerance of *Arabidopsis* by interacting with TEM2 and then co-regulates the salt responsive gene Cor15A (Wang et al., 2014). More recently, Zheng et al. (2016) reported the wheat GT Factor TaGT2L1D negatively regulated osmotic stress tolerance and plant development. Song et al. (2016) and Wang et al. (2016) characterized 20 and 56 full-length trihelix genes in *Chrysanthemum* and *Populus trichocarpa*, respectively. Expression profiling analysis of these genes indicated most of them were significantly induced by abiotic and biotic stress (Song et al., 2016; Wang et al., 2016).

*Brassica napus* is one of the most important oilseed crops in the production of animal feed and vegetable oil. Among the major food crops, Brassica crops are easily affected by drought or osmotic stress and salinity, since they are mainly grown in arid and semiarid areas. Therefore, rapeseeds growth and seed yield have greatly decreased owing to drought and salinity. It’s urgent to develop tolerant *B. napus* cultivars to ensure yield under such adverse conditions. Although great progress has been made in the understanding of the genetic mechanisms of osmotic and salt stress tolerance in the model plants *Arabidopsis* and rice (Nakashima et al., 2009; Fukao and Xiong, 2013), studies on the molecular mechanisms of stress tolerance in Brassica crops have made few advances. In recent years, the major advancements in next-generation sequencing technology have accelerated the batch identification of abiotic response genes. Several studies have identified hundreds of genes that are differentially expressed in response to osmotic or salt stress treatment using RNA-seq and proteomic methods, however, only a very few candidate genes were confirmed to function in salt or osmotic stress tolerance by forward and reverse genetic approaches (Zhang et al., 2014).

In this study, we characterized a new gene, *BnSIP1-1*, which belongs to the SIP1 subfamily of the trihelix transcription factor family. SIP1 clade was named after the first member of this subfamily which was identified through being bound to the *Agrobacterium tumefaciens* 6b oncogenic protein, and was named SIP1 (Kitakura et al., 2002; Kaplan-Levy et al., 2012). The new gene in our study is the first SIP1 clade gene identified in *B. napus*, so we named it as *BnSIP1-1.* The expression of *BnSIP1-1* was induced by osmotic stress, salt and ABA. Interesting, overexpression of *BnSIP1-1* enhanced the plants tolerance to osmotic and salt stress during the germination period, by contrast, during the seedling period, the transgenic plants exhibited only osmotic stress tolerance but not salt tolerance. In addition, our data also revealed that *BnSIP1-1* functions in ABA synthesis and signaling. To date, information about the involvement of the *B. napus* trihelix gene in abiotic response is scarce. Here, we firstly investigate abiotic stress response and possible function mechanisms of a new trihelix gene in *B. napus*.

## Materials and Methods

### Plant Materials

*Brassica napus* cv. ZhongShuang 6 (ZS6), which is an elite Chinese cultivar, and the *B. napus* transgenic plants were grown in Pindstrup soil mix (Denmark) or 1/2 MS medium in a greenhouse at 22°C with 16 h light/8 h dark cycle, 40–65% humidity, and light intensity of 8,000 lux. Mature seeds of these plants were collected for experimental use.

### Cloning and Sequence Analysis of *BnSIP1-1*

The full-length *BnSIP1-1* cDNA was isolated by PCR amplification of cDNA prepared from the mRNA of ZS6 leaves. The primers were listed in Supplementary Table S1. The resulting sequences and the predicted BnSIP1-1 protein sequence were analyzed using BLAST software^1^. The protein structure regions were predicted by PSIPRED 3.3^2^ (Sadowski and Jones, 2009) and the multiple protein alignment was performed using CLUSTALW2^3^ (Li et al., 2013).

### Transient Transformation and Fluorescence Microscopy Observation

The coding sequence of *BnSIP1-1* was amplified using primers *BnSIP1-1*(3; Supplementary Table S1) for transient expression in onion epidermal cells. The PCR products were subcloned into PUC-35S-GFP expression vector under the control of the CaMV 35S promoter. The PUC-35S-GFP vector was transiently expressed in onion epidermal cells using a gene gun (PDS-1000, Bio-Rad, USA). Fluorescence was observed using a fluorescence microscopy (DM2500, Leica, Germany) after incubation at 25°C for 18 h on MS medium. Fluorophores were excited using an argon laser at 488 nm (GFP), and bright-field images were collected using a transmitted light detector.

### Stable Transformation and Generation of Transgenic Plants

To generate the transgenic plants, the CDS of *BnSIP1-1* was amplified from cDNA isolated from ZS6 leaves using *BnSIP1-1*(2) primers (Supplementary Table S1) by reverse transcription PCR, and then subcloned into the PBI121S vector under the CaMV 35S promoter and a terminal poly A sequence (Liu et al., 2014). The 35S:*BnSIP1-1* recombinant vector was introduced into *Agrobacterium tumefaciens* GV3101 by electroporation, and positive clones were selected on LB agar plates at 28°C, supplemented with appropriate concentrations of antibiotics (gentamicin 50 mg L^-1^, rifampicin 50 mg L^-1^ and kanamycin 50 mg L^-1^) and verified using PCR. A single positive colony was used to transform *B. napus* (ZS6). The transgenic *B. napus* plants were generated by *agrobacterium*-mediated method as previously reported (Liu et al., 2014). In total, there were 22 independent T_0_ generation of transgenic lines generated, and 77% were positive transformants as confirmed by PCR with a forward primer designed from the CaMV 35S sequence and a reverse primer designed from the *BnSIP1-1* sequence. Positive T_3_ generation transgenic plants were grown for phenotypic identification.

### Phenotype Identification

For the salt, osmotic or ABA stress tolerance assay of the transgenic *B. napus* during germination period, the wild-type and T_3_ transgenic *B. napus* seeds were planted on plates filled with three-layer filter paper containing 8 ml of mannitol (400 and 600 mM), NaCl (150 and 200 mM), ABA (50 μM ABA and 100 μM ABA) or distilled water without any additions for 4 days. The germination rate was calculated every 12 h. Observations were made and images were acquired using a camera.

For the salt and osmotic stress assay of the transgenic *B. napus* during seedling period, the wild-type and transgenic seeds were germinated on wet filter paper for 7 days, and then transferred to liquid 1/2 MS medium under a 16 h light/8 h dark cycle at 25°C for 8 days. Finally, the 15-day-old seedlings were transferred to liquid 1/2 MS supplemented medium with NaCl solution (150 or 200 mM) or mannitol solution (300 or 600 mM). The control seedlings were transferred to liquid 1/2 MS medium supplemented without any additions. Photographs were taken after 6 h of treatment with 600 mM mannitol and 9 days of treatment with 300 mM mannitol. The chlorophyll contents of the wild-type and transgenic seedlings were detected after 9 days of treatment with 300 mM mannitol. Total chlorophyll was isolated using an extraction solution (2:1 acetone:95% alcohol), and the chlorophyll a and b contents were measured at 646.6, 663.6, and 750 nm using a spectrophotometer (Lambda, PerkinElmer, USA) according to previously described methods (Porra et al., 1989; Mishra et al., 2008).

For the ABA sensitivity assay during seedling period, the wild-type and transgenic *B. napus* seeds were sown in liquid 1/2 MS medium or soil with or without 1 μM ABA or 10 μM ABA. Photographs were taken after 12 days of plating on liquid 1/2 MS medium or 6 days of sowing in soil (Travaglia et al., 2010). The length of stem and root, and fresh weight of aboveground part and root of the wild-type and transgenic plants were measured after 12 days of treatment.

### Real-Time Pcr Assay

RNA extraction and the first-strand cDNA synthesis were performed using an RNAprep Pure Plant Kit (TIANGEN BIOTECH, Beijing, China) and FastQuant RT Kit (with gDNase; TIANGEN BIOTECH, Beijing, China) according to the manufacturers’ instructions. Real-time PCR was performed using fivefold diluted cDNA and a SYBR Green I kit (Bio-Rad, Hercules, CA, USA) on the CFX96 real-time PCR platform (Bio-Rad, Hercules, CA, USA). Three independent biological replicates and three technical replicates for each biological replicate were run and the significance was determined through *t*-test of SPSS statistical software (*p* < 0.05). The *B. napus* β*-actin* gene (accession No. AF111812.1) was used as an internal reference control. Quantitative analysis was performed using the ΔΔCt comparative method (Livak and Schmittgen, 2001).

For the tissue specific analysis, the roots, leaves, and stems were harvested from randomly selected 2-month-old plants; and the pollens, stigmas, petals and immature seeds were collected from plants during reproduction period. For exploring the response of *BnSIP1-1* to different abiotic stresses, the leaves and roots of 15-day-old seedlings (under 50 μM ABA, 200 mM NaCl, or 300 mM mannitol for 0, 1, 2, 4, 8, 12, and 24 h of treatments) were harvested for QPCR analysis. For detection of *BnSIP1-1* expression of transgenic *B. napus*, the leaves of different independent transgenic lines were harvested. The leaves of 15-day-old transgenic and wild-type seedlings (under normal condition or treated with 50 μM ABA, 200 mM NaCl, or 300 mM mannitol for 0, 1, 6, 48, and 72h) were harvested for detection of the expression of stress-related genes (*BnRD29A*, *BnERD15*, *BnLEA1*, *BnNAC485*, *BnCIPK6*, *BnSOS1*, *BnNHX1*, *BnKIN1*, *BnHKT*, and *BnABI5*). The corresponding specific primers were listed in Supplementary Table S1.

### Determination of ABA Content

For ABA content measurement, the leaves of 15-day-old transgenic and wild-type seedlings (under normal condition or treated with 50 μM ABA, 200 mM NaCl, or 300 mM mannitol for 0, 1, 6, and 24 h) were harvested. Quantification of endogenous ABA was performed as described (Chen M. et al., 2012).

### Statistical Analysis

All of the expression, phenotypic and quantitative experiments have been replicated three times and data have been presented as mean ± SD (standard deviation). Significant variations between genotypes or treatments were evaluated statistically by Student’s *t*-test or one-way ANOVA (Sokal and Rohlf, 1995). Mean values that were significantly different (*p* < 0.05) from each other are marked with asterisks or different lowercase letters inside the figures.

## Results

### Sequence Analysis of *BnSIP1-1*

**FIGURE 1:**
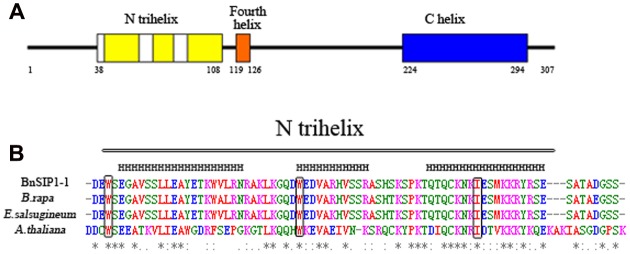
**Conserved domain in the deduced BnSIP1-1 protein. (A)** Structure of the deduced BnSIP1-1 protein of *Brassica napus*. The trihelix DNA binding domains are shown in yellow. The fourth amphipathic a-helix is shown in orange. The long, uninterrupted α-helical sequence in the C terminus is shown in blue. **(B)** Amino acid alignments of the conserved domain trihelix in BnSIP1-1 with orthologous sequences from *B. rapa* (accession No. XP_009116124), *Eutrema salsugineum* (accession No. XP_006403581.1) and *Arabidopsis thaliana* ASIL1 (accession No. NP_564648.1). The conserved tryptophan (or isoleucine) in each helix is indicated by the boxes. The asterisks denote the conserved amino acids.

The full-length gene sequence of *BnSIP1-1* was amplified from *B. napus* to analyze the structural properties. This analysis suggested that *BnSIP1-1* has only one exon. The ORF of *BnSIP1-1* is 924 bp long and encodes a predicted protein of 307 amino acids. Its predicted molecular mass and isoelectric point were 34.6 kDa and 9.30, respectively. Similarity searches revealed that *BnSIP1-1* has 100% overall amino acid identity with a predicted gene product having an unknown function (accession No. CDX73485) in *B. napus*, 96% identity with an uncharacterized protein (accession No. XP_009116124) from *B. rapa*, 83% identity with an unknown protein (accession No. XP_006403581.1) from *Eutrema salsugineum*, and 26 and 19% identity with *Arabidopsis* ASIL1 (accession No. NP_564648.1) and ASIL2 (accession No. OAP02717.1), respectively. Analyses of the helix structure of the predicted protein suggested BnSIP1-1 protein had the classical conserved secondary structure of the SIP1 clade of the trihelix family, including the N-terminal trihelix domain, the fourth amphipathic α-helix, and the long, uninterrupted α-helical (**Figure 1**). Moreover, the three conserved amino acids in the three individual amphipathic α-helices of the N-terminal trihelix were also observed in *BnSIP1-1*, including two tryptophans (W) and one isoleucine (I). Overall, these results suggested that the *BnSIP1-1* gene isolated in this study was a member of the SIP1 clade of the trihelix family.

### Subcellular Localization of the BnSIP1-1 Protein

**FIGURE 2:**
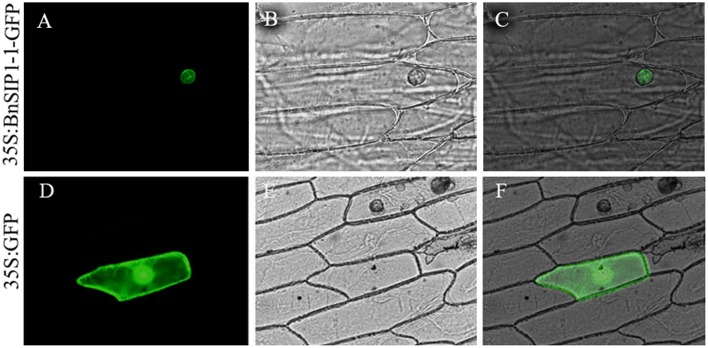
**Subcellular localization of the BnSIP1-1 protein**. Onion cells were transformed with the 35S:BnSIP1-1-GFP **(A–C)** and 35S:GFP **(D–F)** constructs. **(A,D)** Nuclear localization of BnSIP1-1-GFP and GFP photographed in the dark for green fluorescence. **(B,E)** The same cells as in **(A,D)** with bright light. **(C,F)** The merged images of **(A,B)**, and **(D,E)**, respectively. GFP and BnSIP1-1-GFP fusion proteins were under the control of the CaMV 35S promoter.

To further confirm the localization of the *BnSIP1-1* gene product, the *BnSIP1-1* gene was cloned the downstream of the CaMV (constitutive Cauliflower mosaic virus) 35S promoter of pCAMBIA1301-GFP vector and upstream of the *GFP* gene to create the 35S:*BnSIP1-1-GFP* fusion construct. The positive colonies were identified by PCR (**Supplementary Figure S1**). The BnSIP1-1-GFP fusion protein was transiently expressed in onion epidermal cells. Confocal microscopy analysis revealed that the *BnSIP1-1* gene product was localized exclusively in the nucleus of cells, whereas the GFP control was found throughout the entire cell (**Figure 2**).

### *BnSIP1-1* Is Ubiquitously Expressed in *B. napus* Tissues

**FIGURE 3:**
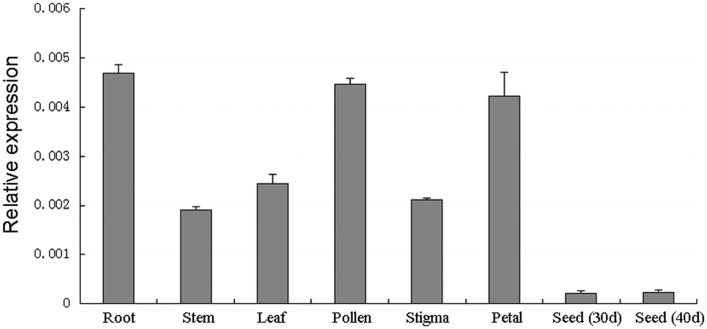
**Expression patterns of *BnSIP1-1* in different tissues in *B. napus* using QPCR analysis**. The *y*-axis represents the relative fold difference in mRNA level and was calculated using the ΔΔCt method with *BnACTIN* as the internal control. The vertical bars indicate ± SD of four replicates on one sample. Three biological experiments were performed, which produced similar results.

The trihelix family genes play important roles in different development processes and growth conditions (Kaplan-Levy et al., 2012). To determine the expression pattern of *BnSIP1-1* in different *B. napus* tissues, QPCR was conducted using mRNA which was isolated from different tissues of the wild-type. The results showed that *BnSIP1-1* was expressed in all of the examined tissues, including the roots, stems, leaves, pollens, stigmas, petals and immature seeds, with high expression levels in the roots, leaves, pollen and petals (**Figure 3**).

### ABA, Salt and Osmotic Stress Induce the Expression of *BnSIP1-1*

**FIGURE 4:**
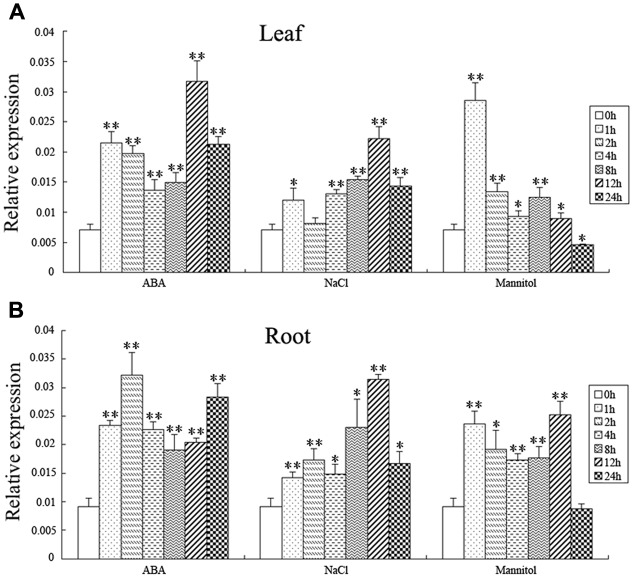
***BnSIP1-1* gene expression pattern in the leaves (A)** and roots **(B)** in the 15-day-old *B. napus* seedlings. *B. napus* plants were treated with 50 μM ABA, 200 mM NaCl, or 300 mM mannitol for 0, 1, 2, 4, 8, 12, and 24 h. The asterisks ^∗^ and ^∗∗^ indicate that the value of a *t*-test was *P* < 0.05 and *P* < 0.01, respectively, and a significant difference between the different abiotic treatments and the control.

In order to explore the response of the *BnSIP1-1* gene to various abiotic stresses, QPCR was used to analyze the *BnSIP1-1* gene expression pattern in leaves and roots of 15-day-old seedlings under ABA, NaCl, and mannitol treatments (**Figure 4**). As shown in **Figure 4A**, the *BnSIP1-1* transcript was remarkably enhanced in the leaves by ABA treatment, which amounted to an approximate threefold increase at the 1 h time point relative to the untreated control plants and reached the highest expression (an approximately fourfold increase) after 12 h of ABA treatment. Under NaCl treatment, *BnSIP1-1* gene expression in the leaves increased gradually and also showed the highest level of expression (an approximately threefold increase) at the 12 h time point. By contrast, *BnSIP1-1* gene expression in the leaves under mannitol treatment showed a rapid and transient approximately fourfold increase at the 1 h time point and then declined over time. The *BnSIP1-1* gene expression eventually was 40% lower than the control plants after 24 h of osmotic stress treatment. The expression pattern of *BnSIP1-1* was similar in the roots as in the leaves under salt stress. However, the expression pattern of *BnSIP1-1* varied in roots under ABA and mannitol stress. *BnSIP1-1* expression in the roots was up-regulated and reached its highest level (an approximately threefold increase) after 2 h of ABA treatment. Under mannitol treatment, the expression of *BnSIP1-1* in the roots was comparatively higher in the first 12 h of treatment and then decreased to a similar level as the control plants after 24 h of treatment.

### Overexpression of *BnSIP1-1* Conferred Osmotic Stress Tolerance in Transgenic *B. napus* Plants

**FIGURE 5:**
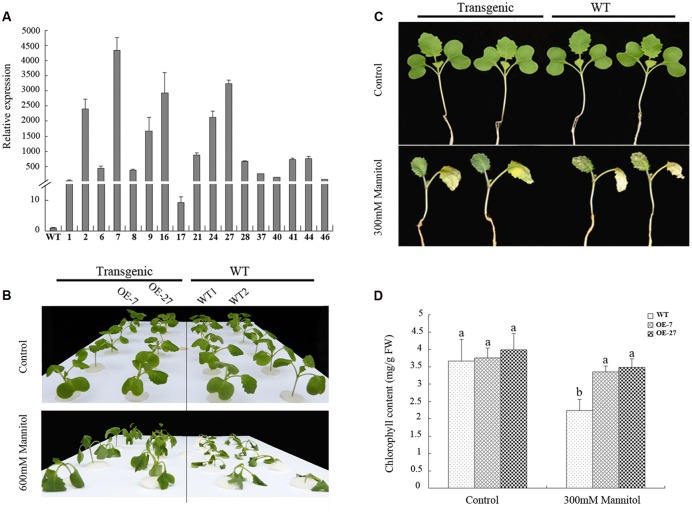
**Overexpression of *BnSIP1-1* in transgenic *B. napus* plants enhanced resistance to osmotic stress. (A)** Relative gene expression of *BnSIP1-1* in the leaves of 2-month-old wild-type and T_0_ 35S:BnSIP1-1 transgenic plants by QPCR. Standard errors were derived from three repeated experiments for the expression levels of each transgenic line. **(B)** Fifteen-day-old seedlings were transferred to 1/2 MS medium supplemented with or without 600 mM mannitol for 6 h before the images shown were taken. **(C)** Fifteen-day-old seedlings were transferred to 1/2 MS medium supplemented with or without 300 mM mannitol for 9 days before the images shown were taken. **(D)** Total chlorophyll contents of the 300 mM mannitol-treated or untreated leaves from **(C)**. FW, fresh weight. Values are mean ± SD (*n* = 3). Means denoted by the same letter did not differ significantly at *P* < 0.05.

To determine whether *BnSIP1-1* played a role in the abiotic stress response, 17 independent transformants of the *B. napus* that OE *BnSIP1-1* under the control of the CaMV 35S promoter were generated. Total RNA from young leaves of independent transgenic lines was extracted for QPCR. The results showed that the *BnSIP1-1* gene was highly expressed in all of the positive lines (**Figure 5A**), and two lines (OE-7, OE-27) were chosen for further analysis. Fifteen-day-old seedlings were treated with salt (150 mM or 200 mM NaCl) or osmotic (300 mM or 600 mM mannitol) stress to analyze their tolerance to abiotic stresses. Compared to the wild-type seedlings, the transgenic lines showed enhanced tolerance to 600 mM mannitol after 6 h of treatment (**Figure 5B**), but no obvious differences were observed after salinity stress (data not shown). The leaves of 15-day-old transgenic seedlings treated with 300 mM mannitol for 9 days looked greener than the wild-type seedlings of the same age (**Figure 5C**). Therefore, we further determined the chlorophyll content of the leaves of the mannitol-treated seedlings. The chlorophyll content of the OE-7 and OE-27 transgenic *B. napus* leaves were 49.6 and 55.6% higher than that of the wild-type leaves, respectively (**Figure 5D**), which indicated that chlorophyll was reduced at a slower rate in the transgenic lines than in leaves of the wild-type seedlings after osmotic stress.

### Overexpression of *BnSIP1-1* Leads to ABA Insensitivity in *B. napus* Seedlings

**FIGURE 6:**
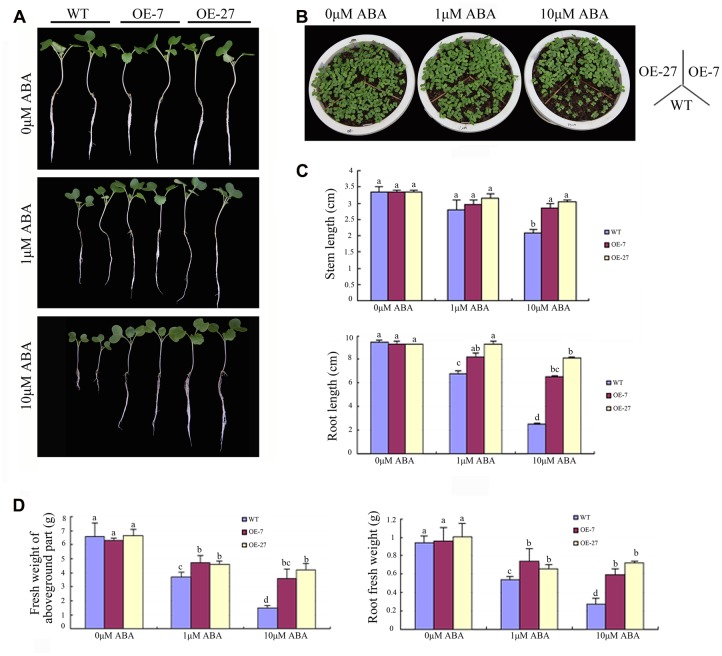
**Reduced sensitivity of the *BnSIP1-1*-OE transgenic *B. napus* lines to ABA. (A)** Growth of the wild-type and *BnSIP1-1* transgenic seedlings on liquid 1/2 MS medium containing 0, 1, and 10 μM ABA. Representative photographs were taken after 12 days of plating. **(B)** Growth of the wild-type and *BnSIP1-1* transgenic seedlings grown in pots containing 0, 1, and 10 μM ABA. Representative photographs were taken after 6 days plating. **(C)** The stem and root lengths of each plant were measured after 12 days plating. **(D)** The fresh weight of the aboveground part and root of each plant was measured after 12 days of plating. Values are mean ± SD (*n* = 3). Means denoted by the same letter did not differ significantly at *P* < 0.05.

The expression pattern of *BnSIP1-1* under ABA and abiotic stresses suggested that it had a significant role in ABA signaling, which prompted us to determine its *in planta* functional characterization. When plated and grown on MS medium, the *BnSIP1-1* transgenic plants grew almost as same as the wild-type plants (**Figures 6A,B**). However, when cultured in MS medium or soil with 1 or 10 μM ABA for several days, the *BnSIP1-1* OE seeds were less sensitive to ABA than the wild-type seeds (**Figures 6A,B**). In the absence of ABA, the root length and weight were similar in the wild-type and transgenic plants. However, under 10 μM ABA treatment, the stems of the transgenic plants were 35–45% longer than those of the wild-type plants (**Figure 6C**). There was no significant difference in the stem length between the wild-type and transgenic seedlings when under 1 μM ABA treatment. The root of the transgenic plants was 22–38% and 160–224% longer than the wild-type plants under 1 and 10 μM ABA, respectively (**Figure 6C**). At the same time, the fresh weight of the transgenic roots was 21–37% and 116–163% higher than the wild-type plants under 1 and 10 μM ABA treatments, respectively (**Figure 6D**). The fresh weight of the aboveground part of the transgenic plants was 24–28% and 142–185% higher than the wild-type plants under 1 and 10 μM ABA, respectively (**Figure 6D**). These data suggested that overexpression of the *BnSIP1-1* gene caused reduced sensitivity to ABA in *B. napus* seedlings.

### *BnSIP1-1* Overexpression Caused Enhanced Germination Rate of *B. napus* under Osmotic, Salt and ABA Stresses

**FIGURE 7:**
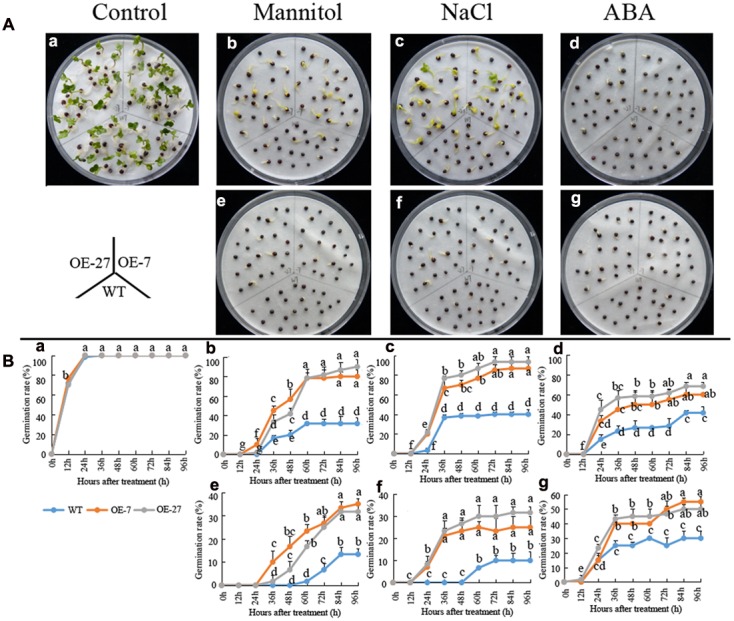
**Germination rates of the wild-type and *BnSIP1-1* OE transgenic seeds under mannitol, NaCl or ABA treatments. (A)** Photographs were taken after 60 h of growth on liquid MS medium containing no addition **(a)**, 400 mM mannitol **(b)**, 150 mM NaCl **(c)**, 50 μM ABA **(d)**, 600 mM mannitol **(e)**, 200 mM NaCl **(f)**, or 100 μM ABA **(g)**. **(B)** The time course of seed germination rates of the wild-type and *BnSIP1-1* overexpression transgenic seeds in liquid MS medium containing no addition **(a)**, 400 mM mannitol **(b)**, 150 mM NaCl **(c)**, 50 μM ABA **(d)**, 600 mM mannitol **(e)**, 200 mM NaCl **(f)**, or 100 μM ABA **(g)**. The data represent the mean ± SD of three independent experiments (germination percentage: 100 seeds for each line). Values are mean ± SD (*n* = 3). Means denoted by the same letter did not differ significantly at *P* < 0.05.

The soil culture experiments of above mentioned dropped a hint that the germination rate of transgenic *B. napus* seeds was higher than that of the wild-type seeds under ABA treatment. To systematically study the effect of *BnSIP1-1* on the germination process, we sowed the wild-type and transgenic T_3_ seeds on plates containing distilled water, mannitol, NaCl, and ABA for 4 days. Without any addition, there were no differences between the wild-type and transgenic seeds. However, when mannitol, NaCl or ABA were applied, the transgenic seeds germinated much better than the wild-type seeds (**Figure 7**). Under lower concentrations of mannitol (400 mM) or NaCl (150 mM), the germination rate of the transgenic seeds could reach higher than 80%, while the germination rate of the wild-type seeds was lower than 40% (**Figures 7A-b/c,B-b/c**) after 96 h of treatment. Under higher concentrations of mannitol (600 mM) or NaCl (200 mM), the germination rate of the transgenic seeds was approximately 30%, while the germination rate of the wild-type seeds was only about 10% (**Figures 7A-e/f,B-e/f**) after 96 h of treatment. In term of the ABA treatment, the transgenic seeds germinated faster than wild-type under both 50 μM ABA and 100 μM ABA treatment (**Figures 7A-d/g,B-d/g**). The increase of ABA concentration had a greater impact on the germination of the wild-type seeds than the transgenic seeds.

### *BnSIP1-1* Overexpression Changed ABA Accumulation

ABA is an extensively studied hormone that plays essential roles in stress responses of plants. Many kinds of abiotic stress promote ABA synthesis, which further induce stomatal closure and up-regulation of the expression of ABA-responsive genes, thereby contributing to plant stress resistance (Finkelstein et al., 2002).

**FIGURE 8:**
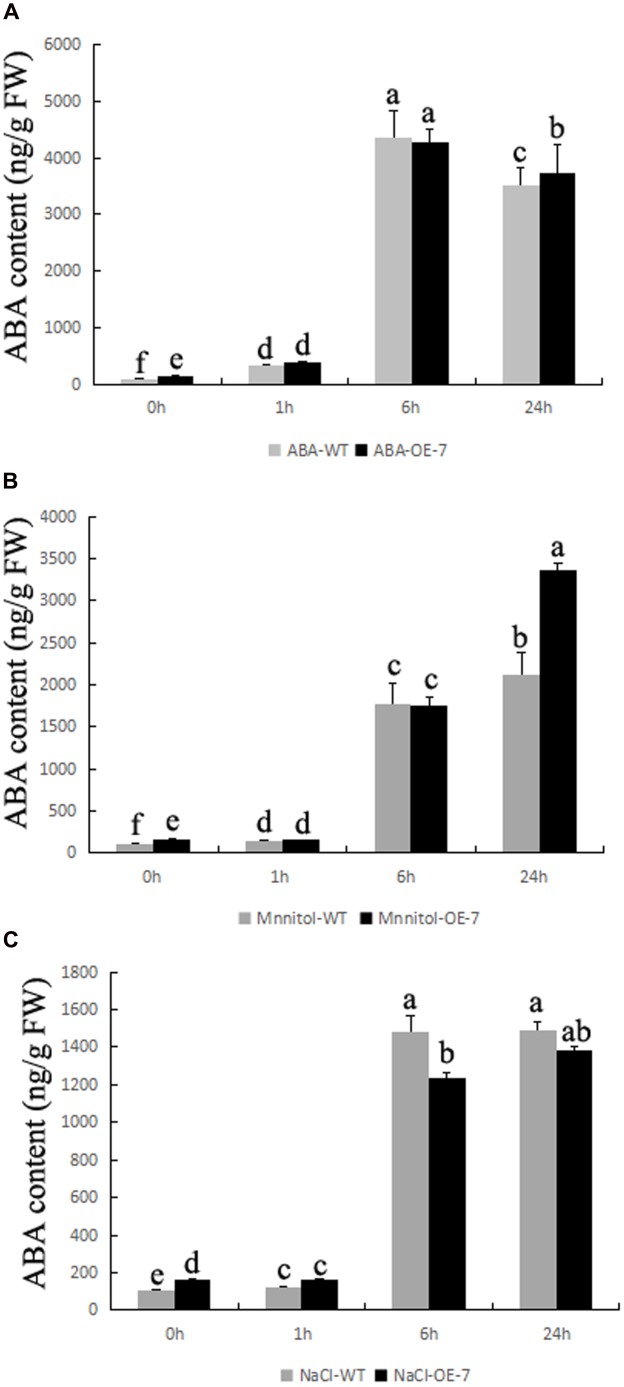
**Changes of ABA content in transgenic plants during stress conditions**. Fifteen-day-old seedlings were transferred to 1/2 MS medium supplemented with 50 μM ABA, 200 mM NaCl, or 300 mM mannitol for 0, 1, 6, and 24 h. **(A)** ABA content of the wild-type and transgenic leaves after ABA treatment for 0, 1, 6, and 24 h; **(B)** ABA content of the wild-type and transgenic leaves after mannitol treatment for 0, 1, 6, and 24 h; **(C)** ABA content of the wild-type and transgenic leaves after NaCl treatment for 0, 1, 6, and 24 h. Values are mean ± SD (*n* = 3). Means denoted by the same letter did not differ significantly at *P* < 0.05.

To study whether OE *BnSIP1-1* affect ABA accumulation, we detected endogenous ABA content in plants (**Figure 8**). All of the stress treatments, including ABA, mannitol and NaCl, can induce significant accumulation of ABA both in the wild-type and the transgenic plants. In addition, endogenous ABA level was 51% higher in transgenic plants than the wild-type during normal growth condition. Under exogenous ABA and mannitol treatment, the accumulation of ABA in the transgenic plants was higher than that in the control plants (**Figures 8A,B**). Under salt stress, the difference of ABA content before treatment was gradually decreased with the prolongation of salt treatment time, then after 24 h of treatment the ABA level in the transgenic plants became similar to the wild-type plants, which showing opposite change trend with ABA or mannitol treatments (**Figure 8C**).

### Altered Expression of Stress-Related and ABA Signaling Genes in *BnSIP1-1* Transgenic *B. napus*

**FIGURE 9:**
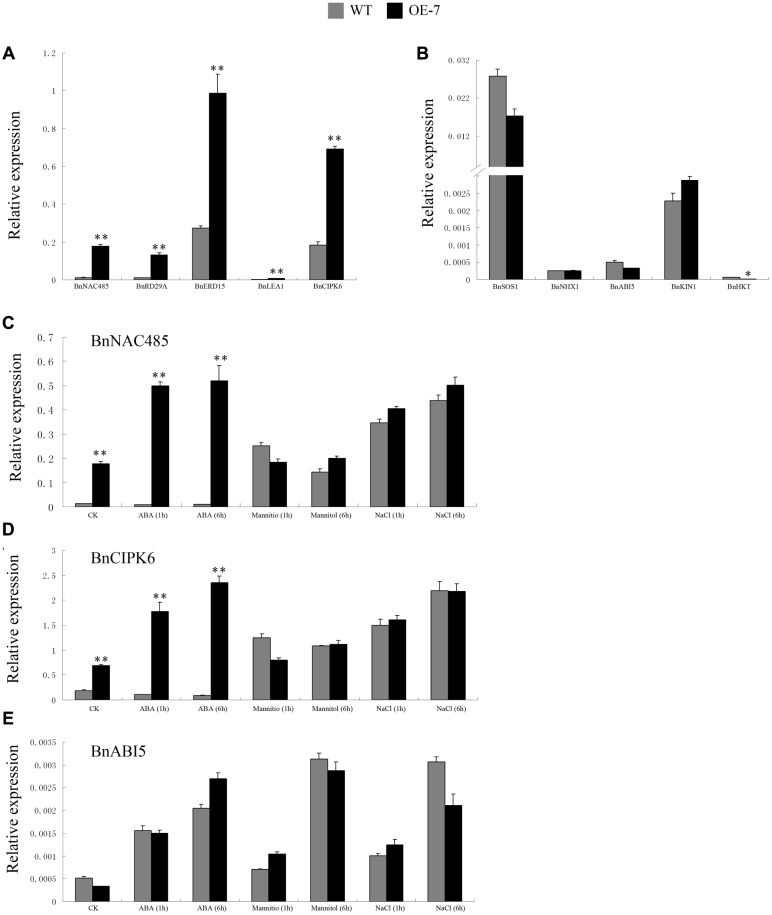
**Expression pattern of stress- and ABA-responsive genes in the transgenic and wild-type plants under normal condition, ABA, mannitol or NaCl treatments. (A)** Transcript abundance of *BnNAC485*, *BnRD29A*, *BnERD15*, *BnLEA1*, and *BnCIPK6* in the leaves of 15-day-old *B. napus* seedlings under normal growth condition. **(B)** Transcript abundance of *BnSOS1*, *BnNHX1*, *BnABI5*, *BnKIN1*, and *BnHKT* in the leaves of 15-day-old *B. napus* seedlings under normal growth conditions. **(C)** Transcript abundance of *BnNAC485* in the leaves of 15-day-old *B. napus* seedlings under control, ABA, mannitol and NaCl treatment for 1 and 6 h. **(D)** Transcript abundance of *BnCIPK6* in the leaves of 15-day-old *B. napus* seedlings under control, ABA, mannitol and NaCl treatment for 1 and 6 h. **(E)** Transcript abundance of *BnABI5* in the leaves of 15-day-old *B. napus* seedlings under control, ABA, mannitol and NaCl treatment for 1 and 6 h. Values are mean ± SD (*n* = 3). The asterisks indicate significant differences (Student’s *t*-test, ^∗^ indicates *P* < 0.05, ^∗∗^ indicates *P* < 0.01).

The *BnSIP1-1* may function in response to stress and ABA through the regulation of downstream genes. To broaden our knowledge about the molecular mechanism of ABA-insensitivity and stress-tolerance in *BnSIP1-1*-OE plants, we first analyzed the expression level of related genes, including *BnRD29A*, *BnERD15*, *BnLEA1*, *BnNAC485*, *BnCIPK6*, *BnSOS1*, *BnNHX1*, *BnKIN1*, and *BnHKT* by QPCR in the wild-type and transgenic plants under normal conditions (**Figures 9A,B**). These genes were categorized into two groups: the expression-increased group (**Figure 9A**) and expression-unchanged/decreased group (**Figure 9B**). As shown in **Figure 9A**, the expression of the transcription factor *BnNAC485* had the most significant increase in *BnSIP1-1* transgenic plants, being approximately 14-fold higher than that in the wild-type plants. Similarly, the expression of *BnRD29A* was increased about 11-fold higher level in the transgenic lines than in the wild-type plants. Furthermore, the expression levels of *BnERD15*, *BnLEA1* and *BnCIPK6* were also up-regulated 3- to 5-fold in the *BnSIP1-1*-OE plants. However, there was no significant difference in the expression levels of *BnSOS1*, *BnNHX1*, *BnABI5* and *BnKIN1* between the transgenic and wild-type plants, as shown in **Figure 9B**. In addition, the expression level of *BnHKT* declined by 68% in the transgenic plants compared with the wild-type under normal growth conditions.

*BnNAC485* and *BnCIPK6* are two rare rapeseed genes which were experimentally identified to play significant roles in ABA signaling and stress responses (Chen L. et al., 2012; Ying et al., 2014). *ABI5* is a basic leucine zipper protein which has already been identified to have important function in the ABA signaling pathway (Piskurewicz et al., 2008). Therefore, to further expand our understanding of regulation mechanism of *BnSIP1-1*, we compared the expression level of *BnNAC485*, *BnCIPK6* and *BnABI5* in the wild-type and transgenic plants treated by ABA, mannitol and NaCl stresses for different time periods. After 1 h of ABA treatment, the expression levels of *BnNAC485* and *BnCIPK6* were about 40-fold and 16-fold higher in the transgenic plants than in the wild-type plants, respectively. As the ABA-treatment time increased to 6 h, the high-expression level of *BnNAC485* remained steady, while the expression level of *BnCIPK6* was further increased to 27-fold in the transgenic plants compared to the wild-type plants. There were no significant differences in the expression levels of *BnNAC485* and *BnCIPK6* under osmotic or salt stresses between the transgenic and wild-type plants. Notably, the expression level of *BnABI5* did not present any difference between the transgenic and wild-type plants under all three of the stress conditions.

## Discussion

Although the trihelix family genes have been shown to function in the developmental processes of plants, their roles in the response to abiotic stress and ABA are largely unknown. Previous studies have shown that some of the gene members in the GT-1, GT-2 and GTγ trihelix subfamilies are involved in the abiotic-stress response, such as *AtGT-3b*, *GmGT-2A*, *GmGT-2B*, and *OsGT*γ*-1* (Park et al., 2004; Xie et al., 2009; Fang et al., 2010). These genes work as positive or negative factors to regulate the adaptability of plants to stresses. In addition, only four genes, *AtGT2L* and *OsGT*γ*-1/2/3* (Fang et al., 2010; Xi et al., 2012), were reported to function in the ABA response. No gene members in the other two subfamilies SH4 and SIP1 have been found to play roles in the abiotic-stress response or ABA response. In the present study, a new SIP1 subfamily gene designated as *BnSIP1-1* from *B. napus* was first identified to be involved in stress responsive and ABA signaling, which could confer stress-tolerance and ABA insensitivity in transgenic *B. napus* plants through the regulation of downstream genes. These studies improve our knowledge about the abiotic stresses and ABA response-related functions of the trihelix family members in plants.

The expression level of *BnSIP1-1* was up-regulated by osmotic and salt stress treatments, indicating a role for the BnSIP1-1 protein in response to abiotic stress. Detailed phenotypic analyses demonstrated that *BnSIP1-1*-OE *B. napus* plants showed a dramatically enhanced germination rate under osmotic or salt stress treatments. In addition, overexpression of *BnSIP1-1* increased the osmotic stress tolerance of the transgenic *B. napus* plants by alleviating seedling wilting and decelerating chlorophyll degradation. Chlorophyll a and b are important photosynthetic pigments in the chloroplast of plant and participate in essential processes of harvesting light energy in the antenna systems by driving electron transfer in the reaction centers (Rissler et al., 2002). The chlorophyll content is generally taken as the primary index of plant stress tolerance (Parihar et al., 2015; Zhou et al., 2016). Overexpressing *SbNHX1* gene could improve the salt tolerance of transgenic *jatropha* and *castor* by depressing deterioration of chlorophyll under different level of salinity stress (Joshi et al., 2013; Patel et al., 2015). Similar result was confirmed by a nuclear-localized histone-gene binding protein from rice (OsHBP1b), playing roles in salinity and osmotic stress tolerance by maintaining chlorophyll content and improving the antioxidant machinery (Lakra et al., 2015). These reports coincided with our findings that the *BnSIP1-1* overexpression transgenic plants had more chlorophyll content and increased tolerance by compared with the wild-type plants under osmotic stress. However, we also detected the response of the transgenic *B. napus* seedlings to salt stress, and no obvious different growth status or chlorophyll content was observed compared to the wild-type seedlings. Therefore, we speculated that *BnSIP1-1* functions differently in response to osmotic and salt stresses through differential regulatory mechanisms during the seedling period.

The plant hormone ABA regulates many key processes in seed dormancy and germination, growth regulation, and stress responses (Ton et al., 2009). Given that the expression of *BnSIP1-1* was induced by ABA (**Figure 4**), we further explored whether *BnSIP1-1* was involved in the ABA-mediated pathway. In terms of seed germination, the transgenic *B. napus* plants showed varying degrees of insensitivity to ABA compared to the wild-type seeds. Furthermore, during the seedling stage, over-expression of *BnSIP1-1* resulted in enhanced growth in the transgenic plant than in the wild-type plants. The phenotype was more obvious under high concentrations of ABA than low concentrations. On this basis, we believe that *BnSIP1-1* negatively participates in the ABA-response pathway.

In addition to the findings noted above, we also drew the conclusion that BnSIP1-1 can regulate the synthesis of ABA (**Figure 8**). ABA levels in the transgenic leaves were higher than those of the control plants under normal condition. Both of the ABA content and synthesis rate in the transgenic leaves were different between different stress conditions. This indicated that *BnSIP1-1* involved in the fine-tune regulation of ABA synthesis. Previous studies have indicated that the induction of the ABA synthesis was conducive to the reduction of water loss, activating stress-related gene expression to improve plant’s osmotic stress tolerance (Sirichandra et al., 2009; Bhaskara et al., 2012). As expected, the higher endogenous ABA content in exogenous ABA-treated and mannitol-treated transgenic plants, and unchanged ABA content in salt-treated transgenic plants were consistent with ABA and osmotic stress tolerance and salt-sensitive phenotype of transgenic plants. Thus, we considered *BnSIP1-1* not only response to exogenous ABA but also regulate endogenous ABA synthesis.

**FIGURE 10:**
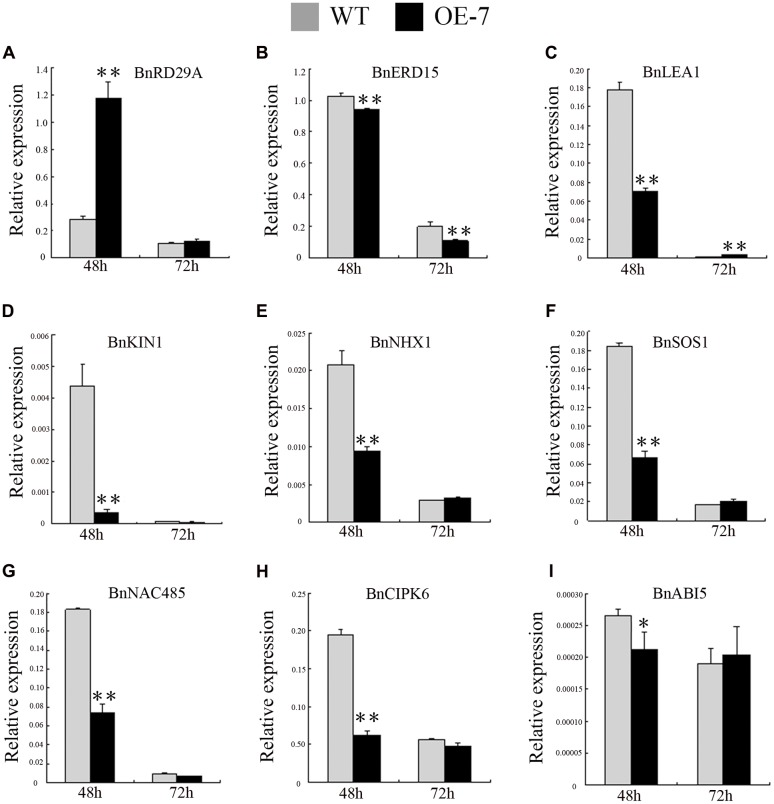
**Expression pattern of stress- and ABA-responsive genes in the transgenic and wild-type plants under 48 and 72 h of NaCl treatments**. Fifteen-day-old seedlings were transferred to 1/2 MS medium supplemented with 200 mM NaCl for 48 and 72 h. Leaves of the transgenic and wild-type plants were harvested for RT-PCR analysis of transcript abundance of **(A)** *BnRD29A*, **(B)** *BnERD15*, **(C)** *BnLEA1*, **(D)** *BnKIN1*, **(E)** *BnNHX1*, **(F)** *BnSOS1*, **(G)** *BnNAC485*, **(H)** *BnCIPK6 and* **(I)** *BnABI5*. Values are mean ± SD (*n* = 3). The asterisks indicate significant differences (Student’s *t*-test, ^∗^ indicates *P* < 0.05, ^∗∗^ indicates *P* < 0.01).

In general, osmotic stress response genes, such as *BnNAC485* and *BnCIPK6*, also can increase the tolerance to salt stress (Chen L. et al., 2012; Ying et al., 2014). However, in the transgenic seedlings, *BnSIP1-1* increased tolerance to ABA and osmotic but not salt stress in our study. We questioned why the *BnSIP1-1* transgenic seedlings had different responses to salt, osmotic and ABA stress. Therefore, we examined the expression of several well-known stress responsive genes, such as *BnRD29A*, *BnERD15*, *BnLEA1*, and *BnKIN1* (Kasuga et al., 1999; Msanne et al., 2011; Dubrovina et al., 2015); ABA-responsive genes, such as *BnNAC485*, *BnCIPK6*, and *BnABI5* (Lopez-Molina et al., 2001; Chen L. et al., 2012; Ying et al., 2014); and salt-responsive genes, such as *BnSOS1*, *BnNHX1* and *BnHKT* (Chakraborty et al., 2012; Dubrovina et al., 2015). Under normal condition, the expressions of *BnRD29A*, *BnERD15*, and *BnLEA1* in the *BnSIP1-1* OE transgenic plants were higher than in the wild-type plants, suggesting *BnSIP1-1* positively regulated the stress response in plants (**Figure 9A**). However, the expressions of salt stress-responsive genes such as *BnSOS1* and *BnNHX*, were unchanged (**Figure 9B**). Moreover, the expression of *BnHKT1* was even decreased in the transgenic plants (**Figure 9B**). *SOS1*, *NHX1* and *HKT* gene are three essential salt-specific response genes, which control Na^+^ exit (*SOS1*) and Na^+^ entry (*HKT*) from the cell, or help the compartmentalization of excess Na^+^ ions in the vacuole (*NHX1*; Zhu, 2003; Joshi et al., 2013; Hamamoto et al., 2014). The expression pattern of the above-mentioned genes in our study illustrated that *BnSIP1-1* perhaps not regulate salt-specific stress-responsive pathway during the seedling period in plants (**Figure 9**). In order to verify our speculation, we further detected the expression of all stress-marker genes at 48 and 72 h of salt treatment to study the long term effect of stress on plants, avoiding the interference of 1/2 MS medium to Na^+^. As shown in **Figure 10**, we noticed that *BnLEA1*, *BnKIN1*, *BnNHX1, BnSOS1, BnNAC485*, and *BnCIPK6* were clearly down-regulated in response to 48 h NaCl treatment. *SOS1* and *NHX1* help cells to remove Na^+^ in cytoplasm to extracellular environment or vacuole under salt stress. The down-regulation of these two genes indicated more toxic Na^+^ accumulation in the transgenic cells cytoplasm after 48 h NaCl treatment, which was consistent with the down-regulation of effector gene, e.g., *BnLEA1* and *BnKIN1*. However, when the processing time was extended to 72 h, the situation was quite different. The effector genes at downstream of stress response pathway *BnRD29A* and *BnKIN1*, salt-specific regulators *BnSOS1* and *BnNHX1*, and ABA response genes *BnABI5*, *BnNAC485* and *BnCIPK6* showed no difference between the transgenic and wild-type seedlings after 72 h of NaCl treatment. We speculated *BnSIP1-1* had a dynamic response to salt stress in the early stage of salt treatment in *B. napus* seedlings. Yang et al. (2014) reported a similar gene, *Cm-BBX24*, which regulated flowering time and drought tolerance, but not salt. In their global transcriptome data, the categories of functional proteins included LEA and dehydration response proteins, members of WRKY, MYB and Zinc finger proteins, and some protein kinases were up-regulated in *Cm-BBX24* overexpression plants. There were also numerous genes related to abiotic stress responses down-regulated, indicating complex function mechanism involved. Therefore, we considered that *BnSIP1-1*, similar to *Cm-BBX24*, did not improve, or at least not strongly improve plant salt tolerance, although they can respond to salt stress. Dynamic measurement of Na^+^ accumulation in the wild-type and transgenic seedlings need to be conducted to further explain why *BnSIP1-1* did not enhance salt tolerance. In addition, Lu Ying et al. reported that overexpression of *BnNAC485* improved the tolerance to osmotic and salt stress and increased the ABA-sensitivity by raising the expression of *ABI5*, which plays an important negative role in the ABA pathway (Piskurewicz et al., 2008; Ying et al., 2014). In our study, the expression level of *BnNAC485* was increased in the *BnSIP1-1* transgenic plants, but ABA hypersensitivity phenotype was not consistent with the *BnNAC485* OE plants. We speculated that *ABI5* played key role for this discrepancy, because the expression of *ABI5* was not significantly changed in the *BnSIP1-1* transgenic plants (**Figure 9E**), while *ABI5* was highly induced in the *BnNAC485* OE plants. Therefore, we considered that the high expression of *BnSIP1-1* inhibited the increase of *BnABI5*, maintaining its expression at a normal level under the control condition and stress treatments and preventing the transgenic plants from responding to the ABA treatment. The above combined analysis indicated that *BnSIP1-1* regulates different pathway genes in response to different stresses through a complex network.

Overall, *BnSIP1-1* can improve seed germination under osmotic, salt or ABA stress treatments, and increase osmotic stress tolerance and reduce ABA sensitivity during the seedling period. The manner in which *BnSIP1-1* regulates downstream genes to respond to different stresses in different developmental processes in plants remains unclear, however, our data clearly illustrated that *BnSIP1-1* played roles in ABA synthesis and signaling, salt and osmotic stress response by regulating *BnABI5*, *BnNAC485* or other stress-related genes. The relationship between the molecular mechanisms of *BnSIP1-1* in response to osmotic, salt and ABA stresses should be further investigated.

## Author Contributions

Jl-L and GW designed the research. ST, FM, JL, XL, XY, XZ, FL, and YW performed the experiments and conducted data analyses. XP edited the manuscript. Jl-L and GW wrote the manuscript. All authors read and approved the final manuscript.

## Conflict of Interest Statement

The authors declare that the research was conducted in the absence of any commercial or financial relationships that could be construed as a potential conflict of interest.

## Footnotes

1 https://blast.ncbi.nlm.nih.gov/Blast.cgi

2 http://bioinf.cs.ucl.ac.uk/psipred/

3 http://www.ebi.ac.uk/Tools/msa/clustalw2

## Supplementary Material

FIGURE S1 **Molecular confirmation of transgenic lines**. PCR amplification of *BnSIP1-1* gene from the wild-type and potential transgenic plants. Lane NC, negative control, lane WT, wild type plant, lanes L, putative transgenic lines and Lane M, marker ladder.

## Abbreviations

ABA: abscisic acid
ANOVA: analysis of variance
CaMV: Cauliflower mosaic virus
GFP: green fluorescent protein
MS: Murashige and Skoog
OE: overexpressing
ORF: open reading frame
QPCR: quantitative reverse transcription–PCR
SIP1: 6b INTERACTING PROTEIN1
ZS6: *Brassica napus* lines cv. ZhongShuang 6.
