# Supplementary figures and images for: BnSIP1-1, a Trihelix Family Gene, Mediates Abiotic Stress Tolerance and ABA Signaling in Brassica napus
Source: Front Plant Sci. 2017 Jan 26;8:44. doi: 10.3389/fpls.2017.00044 (PMC5266734; doi:10.3389/fpls.2017.00044)

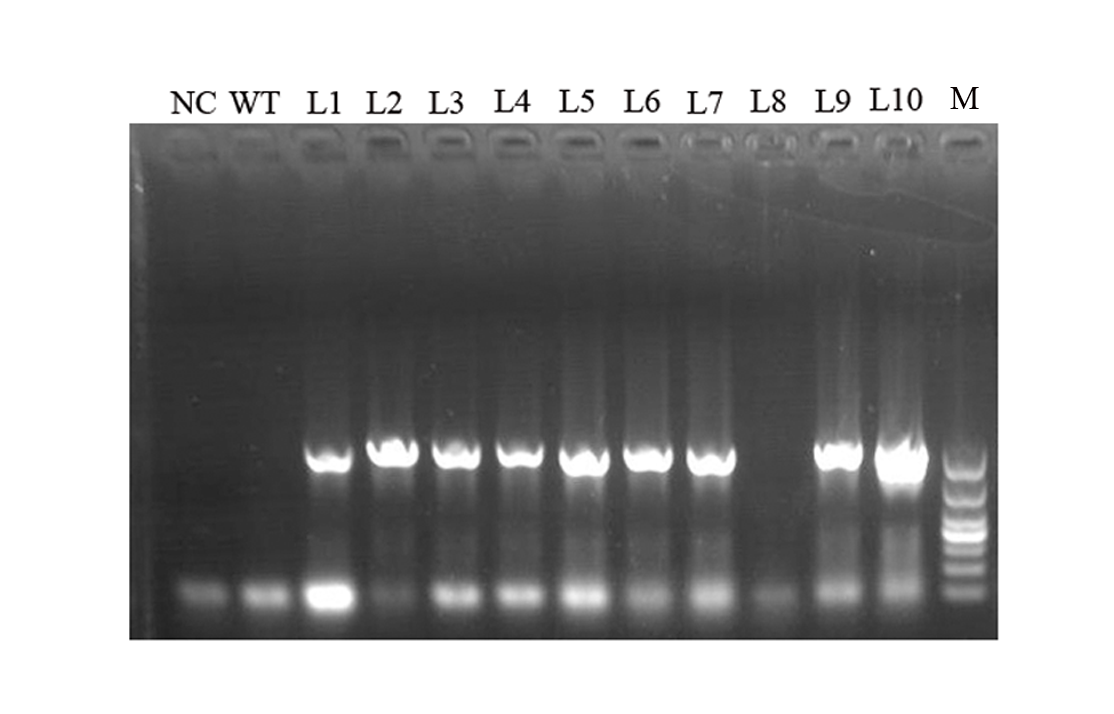

Supplement: FIGURE S1 — Molecular confirmation of transgenic lines. PCR amplification of BnSIP1-1 gene from the wild-type and potential transgenic plants. Lane NC, negative control, lane WT, wild type plant, lanes L, putative transgenic lines and Lane M, marker ladder. [file Image_1.TIF]
