# Supplementary material for: BnSIP1-1, a Trihelix Family Gene, Mediates Abiotic Stress Tolerance and ABA Signaling in Brassica napus
Source: Front Plant Sci. 2017 Jan 26;8:44. doi: 10.3389/fpls.2017.00044 (PMC5266734; doi:10.3389/fpls.2017.00044)
Supplement: Supplementary file 2 [file Table_1.DOCX]

**Supplementary materials**

**Table S1** Primers for isolation and quantitative RT-PCR (QPCR) analysis

Bn: *Brassica napus.*

**Supplementary Table S1**

| **Primer name** | **Sense primers (5′-3′)** | **Antisense primers (5′-3′)** | **Function** |
| --- | --- | --- | --- |
| BnSIP1-1(1) | ATGGAAGACGGAGATTCTAACC | TCAGTTTCTCCCAATCCTTAGTG | cDNA isolation |
| BnSIP1-1(2) | CCCGAGCTCATGGAAGACGGAGATTCTAACC | GCTCTAGATCAGTTTCTCCCAATCCTTAGTG | plasmid construct for stable transformation |
| BnSIP1-1(3) | GCTCTAGAATGGAAGACGGAGATTCTAACC | CCCCCCGGGGTTTCTCCCAATCCTTAGTG | plasmid construct for transient transformation |
| BnSIP1-1(4) | GGAGAGAGCAAGGATGGAG | TCAGTTTCTCCCAATCCTTAGT | Real time PCR |
| BnRD29A | ATTATTGGCTCGGTGGTA | GCTAAGTGGTTGTGATGAC | Real time PCR |
| BnERD15 | AATCGAGTGGGAACAGGAGTC | GAGACTCTGAACATCGTAAAG | Real time PCR |
| BnLEA1 | GTGGTGCCGTATAGTATA | ACAACAGGAAGATCAATG | Real time PCR |
| BnABI5 | GATAACATAGGAGGACAGTAT | CTCAACTACCTTCTCTACC | Real time PCR |
| BnNAC485 | AGACAAGCTGTTGCTTCTCCGGTA | GCCCATTCGAAATTGCCGTTGAGA | Real time PCR |
| BnCIPK6 | GGAGGCGGCGAAGGTTGGGAATAAGTTTGA | GGGCTCGAGTCAAGCAGGTGTAGTAGAAGTC | Real time PCR |
| BnSOS1 | AGACAAGAGCAAGAGTAATC | GATGACAACGAAGATGGT | Real time PCR |
| BnNHX1 | CCTTGCTTGGTGTTGCTA | TCGGTCAGTTGAGTGTCT | Real time PCR |
| BnKIN1 | ATATGCTGCTCCTGAATA | TCTTCCTGATATTAGTTCCA | Real time PCR |
| BnHKT | TCGTGTCTGTATTCTTCTT | ACCAACTCATTCCTTCAA | Real time PCR |
| BnACTIN | CTGGAATTGCTGACCGTATGAG | ATCTGTTGGAAAGTGCTGAGGG | Real time PCR |
